# Ultrasound-Mediated EGF-Coated-Microbubble Cavitation in Dressings for Wound-Healing Applications

**DOI:** 10.1038/s41598-018-26702-z

**Published:** 2018-05-29

**Authors:** Ai-Ho Liao, Chi-Ray Hung, Hang-Kang Chen, Chien-Ping Chiang

**Affiliations:** 1 Graduate Institute of Biomedical Engineering, National Taiwan University of Science and Technology, Taipei, 10607 Taiwan; 2 Department of Biomedical Engineering, National Defense Medical Center, Taipei, 11490 Taiwan; 3 Graduate Institute of Medical Sciences, National Defense Medical Center, Taipei, 11490 Taiwan; 4 Department of Dermatology, Tri-Service General Hospital, National Defense Medical Center, Taipei, 11490 Taiwan; 5 Department of Biochemistry, National Defense Medical Center, Taipei, 11490 Taiwan; 6 Department of Otolaryngology-Head and Neck Surgery, Tri-Service General Hospital, National Defense Medical Center, Taipei, 11490 Taiwan

## Abstract

The feasibility of ultrasound (US) controlled cavitation for transdermal drug delivery (TDD) using gas-filled microbubbles (MBs) has been explored. However, liquid or gel-type MBs is not easy used for TDD. The present study investigated a new treatment model for evaluating the US-mediated liquid-type epidermal growth factor (EGF)-coated lysozyme microbubble (LYMB) cavitation in a wound dressing for enhancing wound healing. The maximum loading efficacy of EGF onto LYMBs was 19.40 ± 0.04%. In terms of the *in vitro* treatment efficacy, the growth of *Staphylococcus aureus* was inhibited by 97.50 ± 1.50% in the group with LYMBs exposed to 3 W/cm^2^ US. During 21 days *in vivo* wound healing experiments, the recovery rate during the first 6 days was significant higher in the group with EGF-LYMB dressings and US exposure (day 6: 54.28 ± 3.26%) than in the control group (day 6: 26.36 ± 3.34%) (*p* < 0.05). Our results show that the new model can significantly reduce the treatment duration during wound healing.

## Introduction

The skin is exposed to the external atmosphere and hence to mechanical, chemical, electrical, thermal, or even nuclear stimuli, which makes it extremely vulnerable to the emergence of different types of lesions such as burns, ulcers, and wounds^1^. The repair of such wounds involves an extremely complex biological and dynamic process consisting of several overlapping phases: hemostasis, inflammation, proliferation, and tissue remodeling^2–5^. Wound healing therapies can be broadly divided into traditional therapies, involving the use of herbal- and animal-derived compounds, living organisms, silver, and traditional dressings, and modern therapies, comprising the use of grafts, modern dressings, bioengineered skin substitutes, and cell/growth factor therapies; these various therapies exhibit distinct levels of efficacy, clinical acceptance, and side effects^6,7^. Modern therapies are generally more expensive than traditional ones, but recent trends are moving toward the development of specialized health-care treatments that involve combining traditional medicine with modern practices and products^1^. The present study integrated a new transdermal drug delivery platform for enhancing wound healing and is the first to determine the effects of US-mediated epidermal growth factor (EGF)-coated lysozyme microbubble (LYMB) cavitation in a wound dressing both *in vitro* and *in vivo*.

The many factors that influence wound healing can be categorized into local and systemic. The characteristics of the wound are directly influenced by local factors^8^. The healing process involves the extracellular matrix, cytokines, blood cells, and growth factors^9^. Cell proliferation through the activation of angiogenesis, myelogenesis, and gene transcription—among other reactions that activate and accelerate the healing process—are stimulated and activated by growth factors^9^. Particularly important factors include the EGF family, platelet-derived growth factor, transforming growth factor beta family, vascular endothelial growth factor (VEGF), basic fibroblast growth factor family, and insulin growth factor^9^. EGF plays an essential role in stimulating cell proliferation, tissue remodeling, and increases in collagen and elastin. VEGF acts on angiogenesis and tissue granulation at the early stage of healing. Platelet-derived growth factor is crucial for inflammation, granulation, re-epithelialization, and remodeling in the three stages of wound healing^8–10^. However, EGF influences the treatment of acute wounds but is limited in chronic wounds due to it readily degrading in the chronic wound environment^11^, but recently EGF in a gel formulation produced increases in the stability, bioactivity, and release of molecules so as to significantly promote and enhance chronic wound healing^10^. Moreover, the topical application of human EGF gel over a wound and wound dressing enhanced wound healing significantly, and was more effective than a conventional povidine iodine dressing. EGF was also found to reduce the number of nonhealing ulcers^12^. A novel polymeric bilayer wound dressing containing EGF-loaded microspheres was synthesized in 2001, and two different doses of EGF were added to prepared gelatin sponges (1 and 15 μg/cm^2^) to activate cell proliferation, with the EGF added either in free form or within microspheres to achieve prolonged release of EGF so as to increase the efficiency^13^. The efficacy and safety of a novel wound dressing composed of hyaluronic acid and collagen containing EGF was evaluated in a clinical trial in 2015^14^.

A previous study found that noncontact, low-frequency ultrasound (US) treatment improved neovascularization and the wound healing in excisional wounds in diabetic mice due to the stimulated release of angiogenic factors^15^. Another study demonstrated that low-intensity therapeutic US can accelerate the formation of fibrin-leukocyte crusts and reduce weight loss in animals with third-degree burns^16^. The findings from that study suggest that the effects of low-intensity therapeutic US are more closely associated with reducing weight loss than accelerating wound healing, and that US is potentially useful as an additional low-cost tool for the treatment of burn injuries. The effects of high-frequency US and high-voltage monophasic pulsed current on the rate of change in the area of chronic and recurrent pressure ulcers in older patients have also been determined^17^. Moreover, low-intensity laser therapy has been considered for repairing diabetic foot ulcers^18^.

Lysozyme (LY) is a naturally occurring enzyme found in bodily secretions such as tears, saliva, and milk. The external application of LY in normal saline at an enzyme concentration of 1 mg/ml was found to shorten the healing time of standard skin wounds in guinea pigs^19,20^. LY loaded into chitosan nanofibers was also found to enhance wound healing due to its antibacterial properties and solubility in water and its synergistic antibacterial effects with EDTA^21–23^. Moreover, LY can promote the healing process, break down bacterial cell walls, and depolymerize chitosan to release *N*-acetyl-d-glucosamine^24^. LY was used in our previous study as the shell of microbubbles (MBs) and combined with US to reduce the dose and treatment duration and improve the prognosis of acne vulgaris^25^. MBs are small gas-filled colloidal particles that are commonly applied in clinical applications as contrast agents for US imaging via intravenous injection. We have recently shown that different conditions of albumin-shelled MBs can enhance their penetration in transdermal delivery *in vivo*^26–28^. However, liquid-type MBs are not easy used for TDD and the survival of MBs with US is affected by the viscosity of the surrounding medium^27^. The present study is the first to integrate EGF-coated LYMBs into a wound dressing and combine them with US as a new platform for enhancing the healing and prognosis of a wounded area both *in vitro* and *in vivo*.

## Materials and Methods

### Preparation and characterization of EGF-LYMB and EGF-LYMB dressings

**Figure 1:**
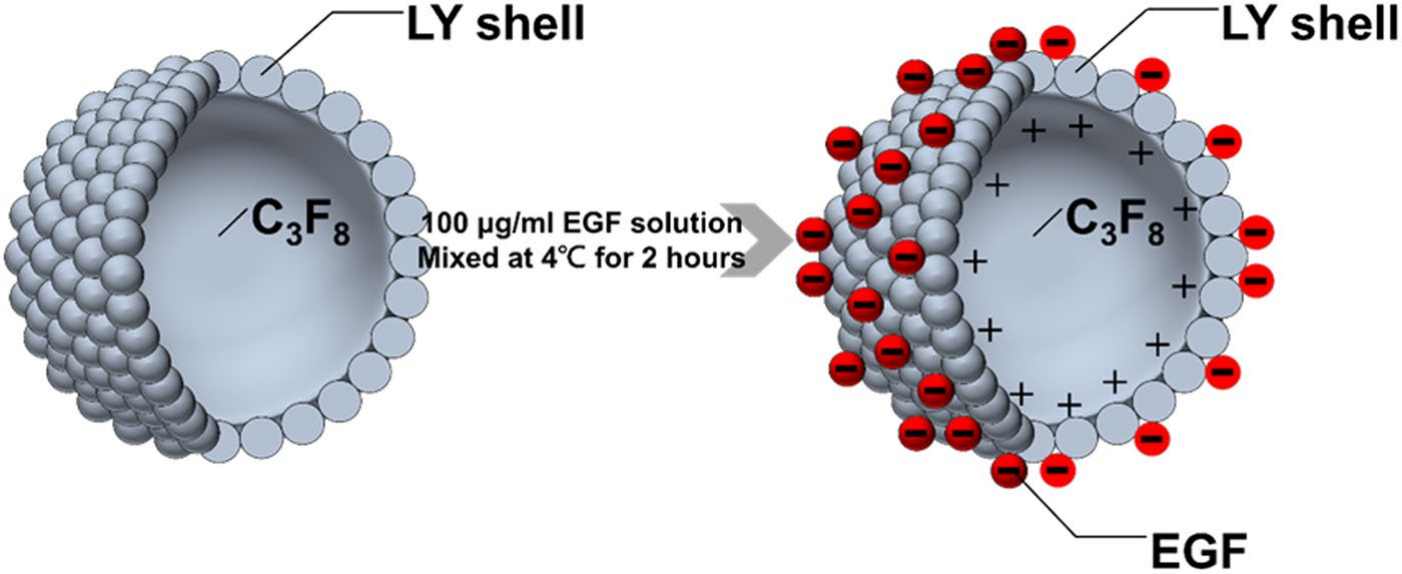
Schematic (not to scale) of the self-assembly of the EGF coating on LYMBs.

Self-assembled EGF-LYMBs were prepared with the composition presented in Fig. 1 and Table 1. Since LY has a positive charge, the surface potential of the LY shell exceeds zero, and thus it can attract molecules with negative charges. Therefore, the LY shell with positive charges can be adsorbed onto EGF that has a negative charge at pH values above 4.6 (which is the isoelectric pH of EGF) by electrical adsorption^29,30^. LYMBs were prepared according to the procedure used in our previous study^25^. In brief, 50 mg of chicken egg-white LY was dissolved in 1 ml of 50 mM Tris buffer (pH 8), and then 20 mg of reducing agent (DL-DTT) was added and the solution was shaken at 50 rpm for 15 min at room temperature to allow sufficient time for partial reduction to occur. MBs were generated by sonicating this solution in perfluoropropane (C_3_F_8_) gas using a sonicator at a power of 120 W (Branson Ultrasonics, Danbury, CT) for 30 s. The MBs were centrifuged at 1200 rpm (128.6 × *g*) for 2 min and then washed three times to eliminate the Tris buffer and DL-DTT using Milli-Q water (pH = 6.4, resistance = 18.2 mΩ). Before incubation with EGF (Recombinant Murine Epidermal Growth Factor, Peprotech, Rocky Hill, NJ), the LYMBs were centrifuged (1200 rpm, 128.7 × *g*; F2402 rotor, Beckman Coulter, Fullerton, CA) for 1 min and then the Milli-Q water was removed. One milliliter of EGF (50, 100, or 200 µg/ml) was then incubated with LYMBs on a rotary shaker (50 rpm; Shaker RS-01, TKS, New Taipei City, Taiwan) for 1 hour at 4 °C in a refrigerator to produce EGF-LYMBs (designated as EGF-LYMB50, EGF-LYMB100, and EGF-LYMB200, respectively). These MBs were washed three times to ensure that the unbound EGF was removed. The number of EGF-LYMBs in the solution was measured with the MultiSizer III device (Beckman Coulter) using a 30-μm aperture probe whose measurement boundary ranged from 0.6 to 20 μm. The size distribution in the suspension was measured using dynamic light scattering (Nanoparticle Analyzer, Horiba, Kyoto, Japan).

**Table 1:** Composition, zeta potential, diameter, and EGF encapsulation efficiency of various LYMBs.

MBs	EGF (µg/ml)	Zeta potential (mV)	Size (µm)	Encapsulation efficiency (%)
LYMBs (original)	n.a.	80.33 ± 1.19	2.62 ± 0.82	n.a.
EGF-LYMB50s	50 µg/ml	53.48 ± 3.32	3.14 ± 0.65	5.84 ± 0.02
EGF-LYMB100s	100 µg/ml	40.33 ± 3.62	3.47 ± 0.73	19.40 ± 0.04
EGF-LYMB200s	200 µg/ml	38.93 ± 2.14	3.51 ± 0.46	9.74 ± 0.06

Data are mean ± SD values.

The morphology of the EGF-LYMBs was characterized by filtering them using a 5-μm syringe filter (Sartorius, Goettingen, Germany) and then hardening them using 0.25% glutaraldehyde (Sigma-Aldrich, St. Louis, IL, USA). To characterize the morphology of the EGF-LYMB wound dressing, 5 μl of 10-fold-diluted EGF-LYMBs was loaded onto a 2 mm × 5 mm dressing. The morphology of the hardened LY-shelled MBs and the EGF-LYMBs dressing was studied using scanning electron microscopy (SEM) after coating the samples with platinum (achieved at 20 mA for 20 min) using an automatic sputter coater (JFC-1300, JEOL, Tokyo, Japan). SEM images were recorded at an accelerating voltage of 15 kV.

### *In vitro* high-frequency US imaging of EGF-LYMBs

**Figure 2:**
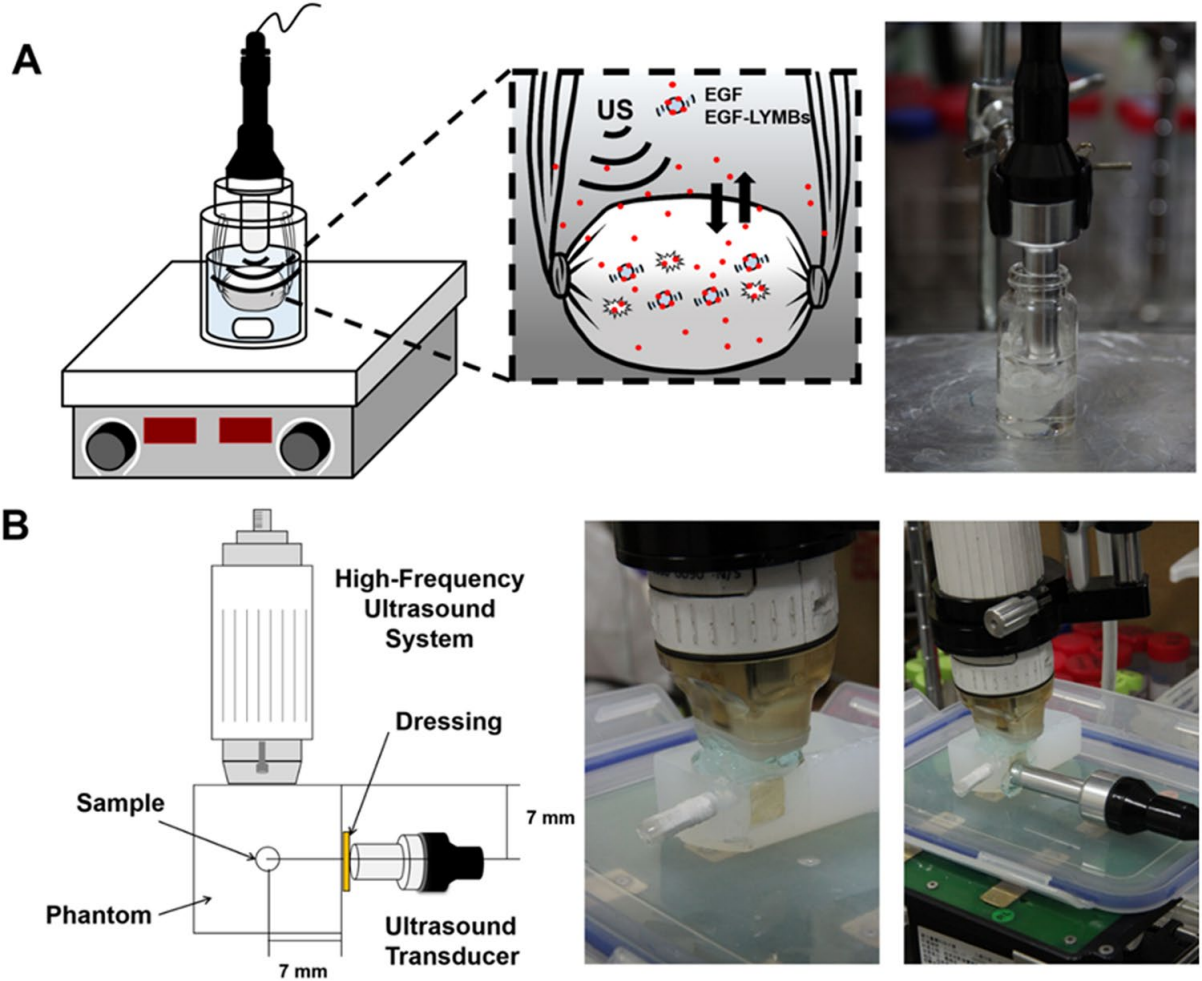
Schematic diagrams and photographs of the *in vitro* release of EGF from LYMBs (**A**) and the US imaging system for EGF-LYMBs loaded in the phantom (**B**).

Since inertial cavitation results in much greater permeability enhancement of the stratum corneum than stable cavitation^31^, high-frequency US imaging was performed to observed bubble disruption using an US animal imaging system (Prospect, S-Sharp Corporation, New Taipei City, Taiwan), as shown in Fig. 2B. The US transmit frequency was 40 MHz, and the transducer had a diameter of 7 mm and a fixed focus at 12 mm. A 2% agarose phantom was constructed with a 2 mm × 2 mm × 20 mm chamber at its center into which to load the EGF-LYMBs. The EGF-LYMBs loaded in the phantom were sonicated with US energy across the dressing (Tegaderm^TM^, 3M Health Care, Neuss, Germany) using the 1-MHz transducer of the sonoporation gene transfection system (ST 2000V, NepaGene) at power densities of 1, 2, and 3 W/cm^2^ for 1 min. The region of interest was drawn over the entire EGF-LYMBs -loaded chamber in a two-dimensional imaging plane by an operator, the dynamic range was set at 50 dB and the average pre- and postsonication image intensities were measured in B-mode images using MATLAB (The MathWorks, Natick, MA, USA).

### Measurements of penetration depth in pigskin

The enhancement of penetration depth by using US-mediated LYMB cavitation in a wound dressing was demonstrated in in pigskin. Fresh porcine skin was obtained from the affiliated slaughterhouse of New Taipei City Meat Market, and all experiments on the skin samples were completed within 6 hours. Circular porcine ear skin samples were constructed with a radius of 1.2 cm and a thickness of 3 mm (encircled with US gel to prevent leakage); the round area of each pigskin sample was loaded with Evans blue or an MB-loaded dressing. Before US treatment, 100 μl of EGF-LYMBs was loaded into the 1-cm^2^ dressing (Tegaderm^TM^, 3M Health Care). The treatment area of the sample was sonicated using the 1-MHz US transducer of the sonoporation system attached to the top of the sample across the dressing, and this was performed successively at power densities of 1, 2, and 3 W/cm^2^ for 1 min. After removing the MB-loaded dressing, 100 μl of Evans blue (0.25% w/w) was added and left for 15 min, and then the area was washed with PBS three times for 1 min each. The treated areas of pigskin were removed and then embedded in an optimal-cutting-temperature solution (Surgipath FSC 22, Leica Microsystems, Buffalo Grove, IL, USA) on round specimen disks with a diameter of 2.2 cm. The embedded samples were placed on the −25 °C freezing stage of a cryostat (Microm HM550 series, Thermo, Braunschweig, Germany) for about 30 min, and transverse sectioning was then performed at a slice thickness of 10 μm. Sections attached to the microscopy slides were air-dried at room temperature and mounted for microscopy examination. The distribution of the Evans blue in the cryosections was determined using an upright microscope (DM 2500, Leica Microsystems). According to the results of high frequency ultrasound imaging and penetration depth in pigskin, the greatest US power density (3 W/cm^2^ for 1 min) was obtained and used for the subsequent experiments involving *in vitro* and *in vivo*.

### Measurement of the EGF on LYMBs

The enzyme-linked immunosorbent assay (ELISA) can be used to efficiently screen the amounts of EGF incorporated into the LY shell of the MBs. Before performing the ELISAs, the EGF-LYMBs were sonicated with US energy at a power density of 3 W/cm^2^ for 1 min to destroy the EGF-LYMBs. The concentrations before and after MB destruction were measured using the MultiSizer III device (Beckman Coulter), which revealed that the sonication process destroyed 95% of the MBs. EGF and EGF-LYMB fragments at various concentrations in 0.04% Proclin 300 (1–5% CMIT/MIT, BOC Sciences, Shirley, NY) were coated onto a mouse EGF 96-well microplate (AssayMax^TM^, St Charles, MO) and incubated for 2 hours at room temperature. The plates were washed five times with washing buffer (200 μl) containing 0.02% sodium azide (Sigma-Aldrich) to remove the excess liquid and fragments as described above. The primary antibody (50 μl; biotinylated mouse EGF antibody, AssayMax^TM^) was then added to each well, and the plate was incubated for 2 hours at room temperature (20–25 °C). The plate was then washed five times, and 50 μl of secondary antibody (streptavidin-peroxidase conjugate, AssayMax^TM^) was added to each well, and the plate was incubated for 30 min at room temperature. The chromogen substrate solution (50 μl of 5% (w/v) 3,3′,5′-tetramethylbenzidine in H_2_O, Sigma-Aldrich) was then added, and the plate was incubated at room temperature for 12 min or until the optimal blue color density had developed. The appropriate stop solution (50 μl of 0.5 N HCl, Sigma-Aldrich) was then added. Colorimetric measurements of avidin were performed at 450 nm using an ELISA reader (Epoch, Biotek, Winooski, VT, USA). A standard EGF calibration curve was constructed to obtain the corresponding concentration of EGF in the EGF-LYMBs based on the measured absorption peaks. Triplicate measurements were performed for each concentration of EGF.

### *In vitro* release

The *in vitro* release behaviors of EGF from MBs were investigated using a dialysis method. Figure 2A shows a schematic of the system setup. Briefly, 1 ml of an EGF-LYMB suspension (having the original concentration after production) was loaded into a dialysis bag with a molecular weight cutoff of 12,000–14,000 and dialyzed against the release media of phosphate-buffered saline (PBS) at pH values of 5.0 and 7.4 within 0.5 °C of 37.0 °C, and with stirring by a magnetic bar at 100 rpm. After 0.5 hours, unfocused US therapy was applied for 1 min using the 1-MHz transducer positioned 3 mm from the top of the dialysis bag under the liquid level at a power density of 3 W/cm^2^ (acoustic pressure = 0.266 MPa). Samples (100 µl) were taken from the release medium after 0.1, 0.2, 0.3, 0.4, 0.5, 1, 2, 3, 4, 5, and 6 hours, with the same volume of PBS added to replace it in the release medium. These samples were kept in a freezer until analyzed using ELISA as described above. The mean values were calculated for four replicate measurements. The drug release profile of EGF was examined as a control. The cumulative release percentage of EGF from MBs was calculated using the following equation^32^:

1 $$R=\frac{{c}_{n}{v}_{0}+{\sum }_{i-0}^{n-1}{c}_{i}{v}_{i}}{W}\times 100 \% $$

where *R* is the release rate, *c*_*n*_ is the drug concentration in the release medium the last time point, *v*_0_ is the total volume of the release medium (10 ml), *v*_*i*_ is the volume of medium withdrawn each time (100 µl), *c*_*i*_ is the drug concentration in the release medium at interval *i*, and *W* is the weight of loaded drug initially^32^.

### *In vitro* treatment efficacy of EGF-LYMBs against *S*. *aureus* colonies

*Staphylococcus aureus* (*S*. *aureus*) cells (BCRC10777, Bioresource Collection and Research Center, Hsinchu, Taiwan) were cultured on nutrient broth (BD Falcon™, Sparks, MD, USA) under anaerobic conditions at 37 °C. To keep the bacterial survival and growth stable, 50 μl of *S*. *aureus* (2 × 10^7^ colony-forming units [CFU]/ml) was added to 3 ml of nutrient broth (0.8 g/100 ml; BD Falcon™) in a sterilized test tube (14-ml polypropylene round-bottomed tube, BD Falcon™). For the antigrowth assay, 500 μl of *S*. *aureus* was adjusted to a concentration to 2 × 10^7^ CFU/ml using the plate count method, mixed with 500 μl of EGF-LYMBs (1.42 × 10^8^ LYMBs/ml, containing 25 mg/ml LY) in an Eppendorf tube, and sonicated by the 1-MHz US transducer of the sonoporation system successively at 3 W/cm^2^ for 1 min. The duty cycle was set at 50%, and a 0.6-cm-diameter US transducer was used. The change in temperature during US sonication at a power density of 3 W/cm^2^ for 1 min at 37 °C did not exceed 0.3 °C, as measured by a thermometer (Optris LS, Optris, Berlin, Germany). The solution was then rested for 1 hour, and the samples were diluted 1:10^5^ in PBS, and 100 µl of each sample was spotted on nutrient agar plates (BD Falcon™). The samples were incubated at 37 °C under anaerobic conditions for 24 hours, and then the CFU of *S*. *aureus* were quantified with the aid of image-analysis software (ImageJ, National Institutes of Health, Bethesda, MD, USA).

### Animal treatments

Six-week-old ICR mice weighing 20–25 g were obtained from Bio Lasco (Taipei, Taiwan). The experimental protocol was approved by the Institutional Animal Care and Use Committee of the National Defense Medical Center, Taipei, Taiwan. Animals were cared for in compliance with institutional guidelines and regulations. Throughout the experiments, the animals were housed in stainless-steel cages in an air-conditioned room with the temperature maintained at 25–28 °C and with alternating light and dark periods of 12 hours each. The animals were acclimatized for 7 days prior to the experiments. Two wound areas with diameters of about 8 mm were created on the dorsal skin of each animal using a dermatome when the animal was 8 weeks old.

The animals were divided into the following five groups with and without US treatment (*n* = 5 per group): (i) no treatment (group C), (ii) wound dressing (1 cm^2^) alone (group D), (iii) 100 µl of LYMBs (1.420 ± 0.011 × 10^8^ LYMBs/ml, mean ± SD) added to the 1-cm^2^ wound dressing (group M), (iv) 100 µl of EGF (20 µg/ml) added to the 1-cm^2^ wound dressing (group E), and (v) 100 µl of EGF-LYMBs (containing 20 µg/ml EGF conjugated on 1.420 ± 0.011 × 10^8^ LYMBs/ml) added to the 1-cm^2^ wound dressing (group EM). The US was applied for 1 min at 3 W/cm^2^ (acoustic pressure = 0.266 MPa). The treatments were applied once daily for 3–4 weeks, and the area of the wound was photographed with a scale by a monocular camera. The wound areas were calculated on each measurement day before and after treatment using MATLAB (The MathWorks) and ImageJ (National Institutes of Health). The wound healing was quantified according to the wound areas using the following equation^33^:

2 $${\rm{Wound}}\,{\rm{healing}}( \% )=\frac{{A}_{1}-{A}_{n}}{{A}_{1}}\times 100 \% $$

where *A*_1_ is the wound area immediately after creating the wound and *A*_*n*_ is the wound area at the measurement time point.

### Image processing for measuring the wound area

**Figure 3:**
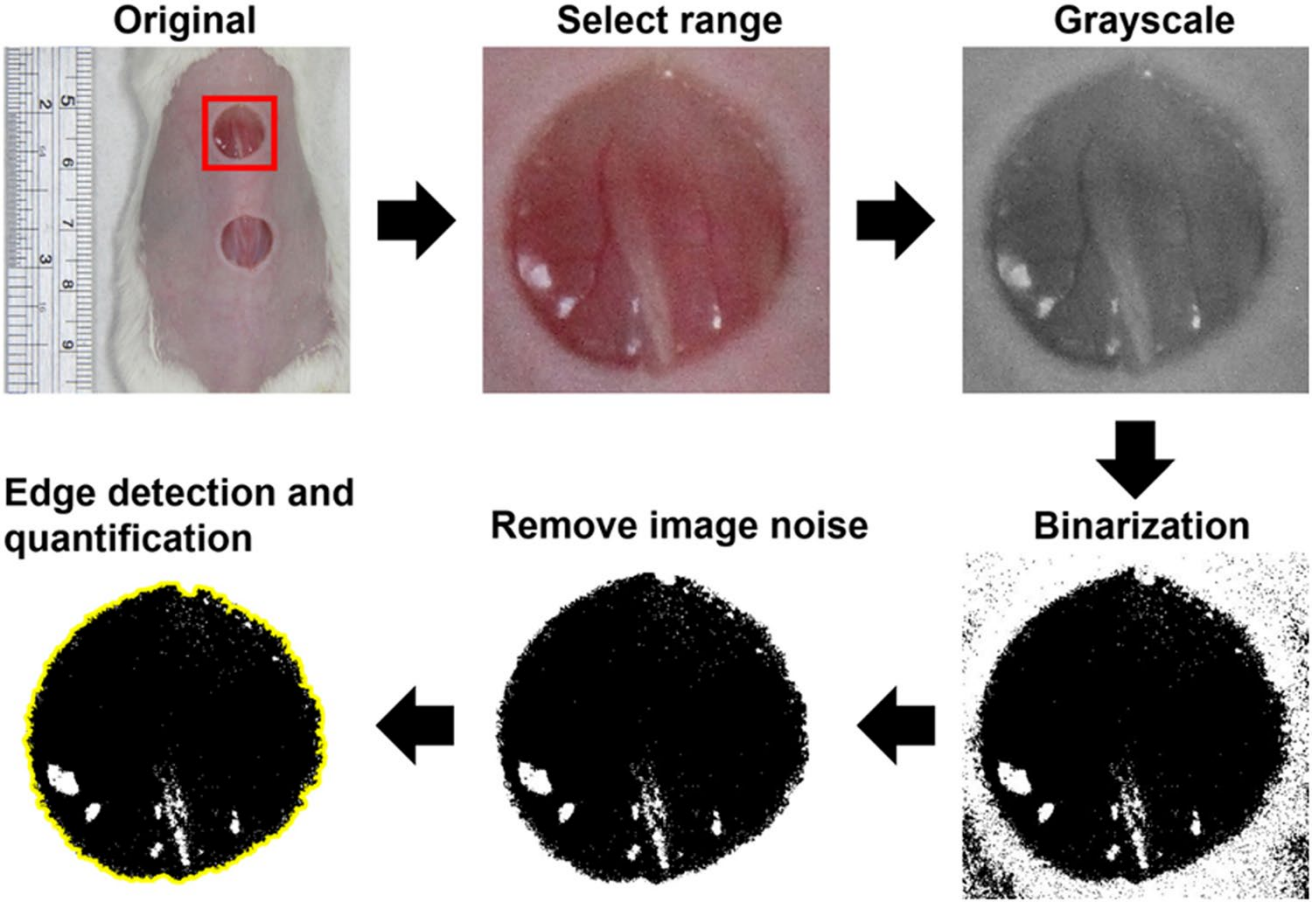
Image-processing procedure for measuring the wound area.

The wound areas were measured using MATLAB (The MathWorks). The image-processing procedure is shown in Fig. 3. The images were first converted from color to grayscale, and image histogram-based binarization was performed using peak-and-valley thresholding applied to the histogram for many experiments^34^ (Sezgin and Sankur 2004). The boundary was then detected using Sobel-operator-based edge detection^35^ (Ahammer and DeVaney 2004), with the same threshold used when processing all of the images. Finally, the wound area enclosed by the detected edge was calculated using the Analyze*-*Set Measurements*-*area and Analyze*-*Measure functions of ImageJ.

### Histochemistry

Skin tissue samples (approximately 8 mm × 8 mm) were cut from the treatment area immediately after the experiments on day 21 and stored in a 10% formalin solution. Hematoxylin and eosin (Sigma-Aldrich) and Masson’s trichrome staining (Sigma-Aldrich) were applied. The samples were then analyzed by an expert histologist.

### Statistical analysis

The obtained data were analyzed statistically using Student’s *t*-test. A probability value of *p* < 0.05 was considered indicative of a significant difference.

## Results

### Preparation and characterization of EGF-LYMB and EGF-LYMB dressings

**Figure 4:**
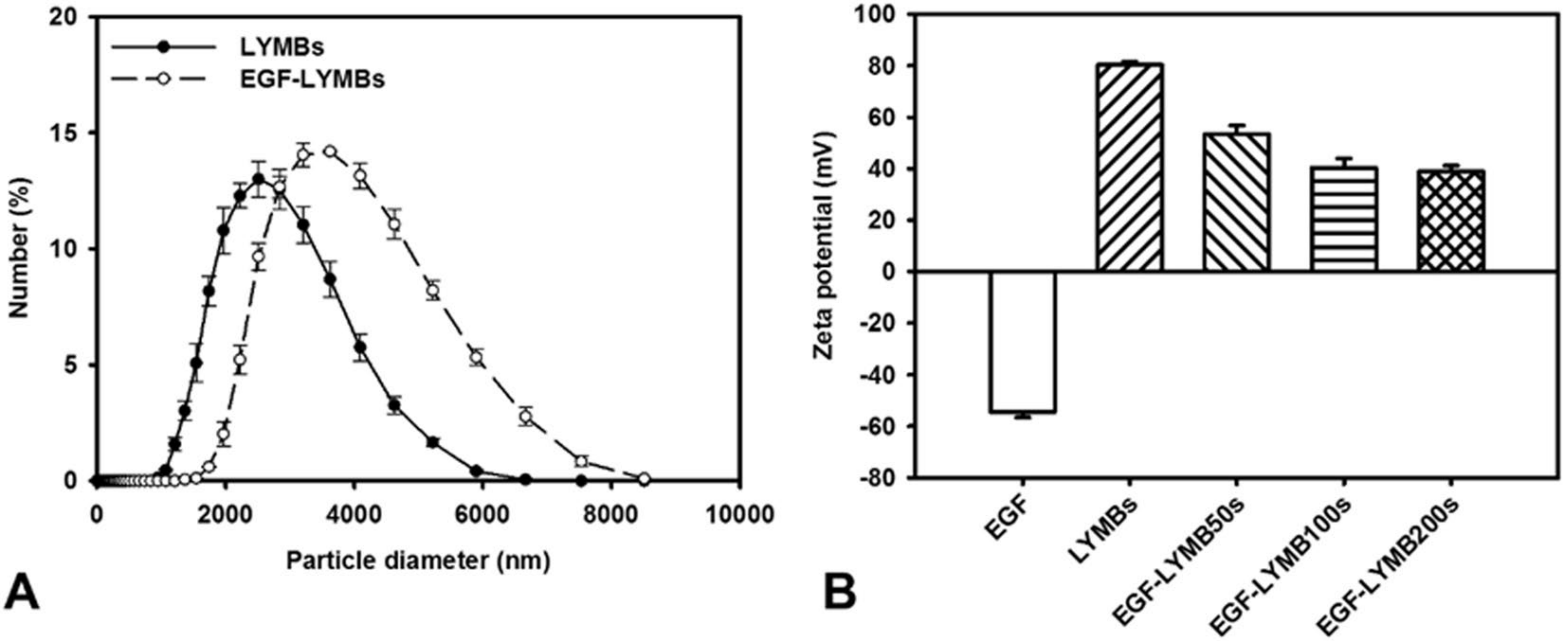
Size distributions (**A**) and zeta potentials (**B**) of LYMBs and EGF-LYMBs. Data are mean and SD values.

The diameters of the LYMBs and EGF-LYMB100s were 2680 ± 210 and 3780 ± 180 nm, respectively (Fig. 4A); the corresponding concentration of LYMBs was 1.42 ± 0.01×10^8^/ml (*n* = 8). The LY content in the original LY-shelled MB solution constructed using a sonicator power of 120 W was 25 mg/ml. The zeta potentials of the EGF, LYMBs, and EGF-LYMBs dispersed in an aqueous solution (pH = 6.4, resistance = 18.2 mΩ) were measured using a Nanoparticle Analyzer (Horiba). LY is a positively charged protein at pH 7, and the LYMBs had a potential of +80.33 ± 1.19 mV. The original potential of EGF (−54.43 ± 2.12 mV) was reversed to surface potentials of EGF-LYMB50s, EGF-LYMB100s, and EGF-LYMB200s of +53.48 ± 3.32, +40.33 ± 3.62, and +38.93 ± 2.14 mV, respectively (Fig. 4B and Table 1) (n = 6).

The encapsulation efficiency (Table 1) of the EGF coated on the LYMB shells was analyzed using the ELISA method, revealing a maximum loading efficiency of EGF-LYMB100s of 19.40 ± 0.04% (*n* = 5). These EGF-LYMBs were used for the subsequent experiments involving *in vitro S*. *aureus* colonies and *in vivo* animal treatments.

**Figure 5:**
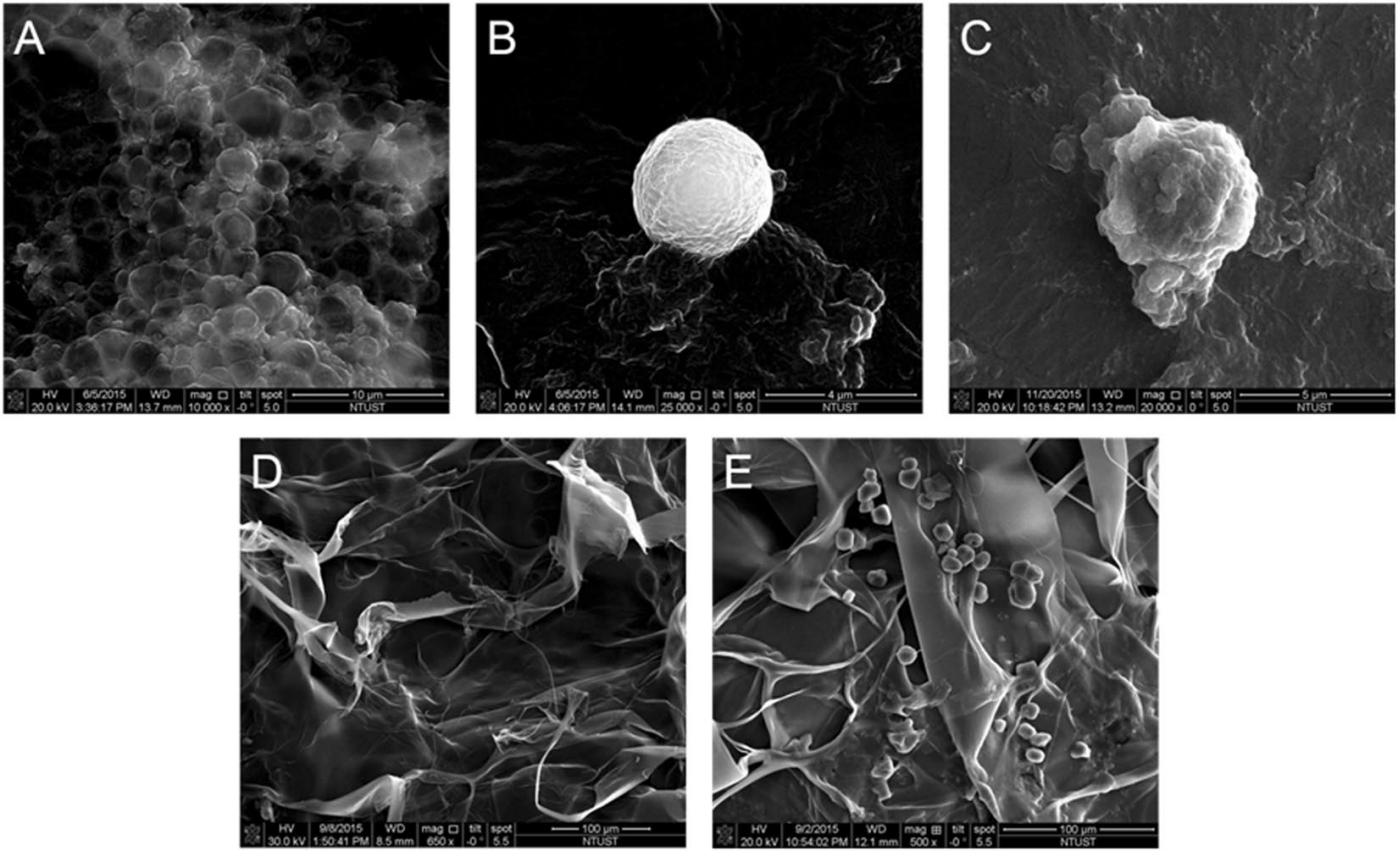
SEM images of LYMBs (**A**), a single LYMB (**B**), a single EGF-LYMB (**C**), a dressing (**D**), and EGF-LYMBs adhering to a dressing (**E**).

Figure 5A–E show SEM images of LYMBs, a single LYMB, a single EGF-LYMB, a dressing, and EGF-LYMBs adhering to a dressing, respectively. The composite structures of the LYMB shell, the EGF-LYMB shell, and the EGF-LYMB shell on a dressing were observable by SEM, which indicated the presence of nanoscale particles after coating with EGF. Figure 5E indicates that the LYMBs interacted and integrated well with the surrounding fibers after being added to the dressing.

### *In vitro* release

**Figure 6:**
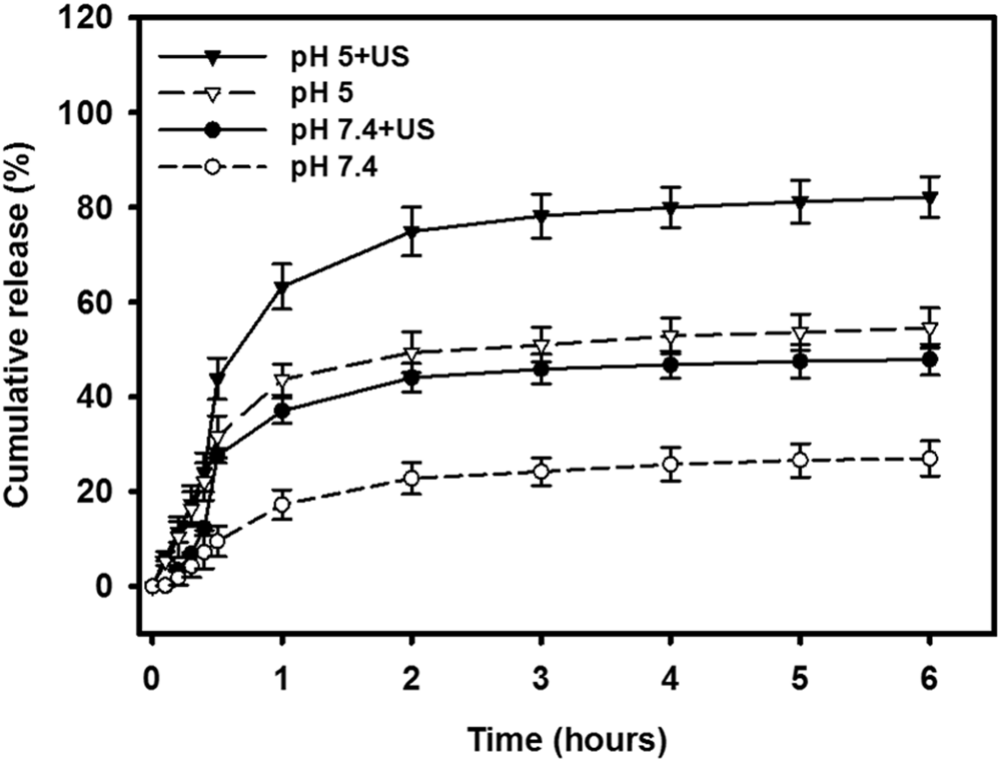
Cumulative drug release of EGF over 6 hours from EGF-LYMBs with and without US sonication in PBS (pH 5 and 7.4). Data are mean and SD values.

Figure 6 shows the percentage cumulative EGF release after 6 hours for EGF-LYMBs and for US combined with EGF-LYMBs (group US + EGF-LYMBs) in PBS for pH values of 5 and 7.4. In the pH-5 environment, 31.5% of the free EGF had diffused through the dialysis membrane relative to control over the first 0.5 hours, and this increased to 43.8% with US sonication. In the pH-7.4 environment, only 9.3% of the free EGF had diffused through the dialysis membrane relative to control over the first 0.5 hours, and this increased to 27.5% with US sonication. Without US sonication, the proportion of the free drug suspension that was released across the dialysis membrane was reduced to only 54.6% at pH 5 and 27.0% at pH 7.4 after 6 hours. With US sonication, the *in vitro* release of the EGF was rapid during the first 2 hours, reaching 74.9% at pH 5 and 44.0% at pH 7.4, followed by a slower but sustained release of EGF from the EGF-LYMBs to just over 82.2% at pH 5 and 47.9% at pH 7.4 after 6 hours. These findings indicate that the application of US energy enhanced drug release by 39.0–195.7%, and also that the pH value affects the efficiency of EGF release from EGF-LYMBs.

### *In vitro* high-frequency US imaging of EGF-LYMBs

**Figure 7:**
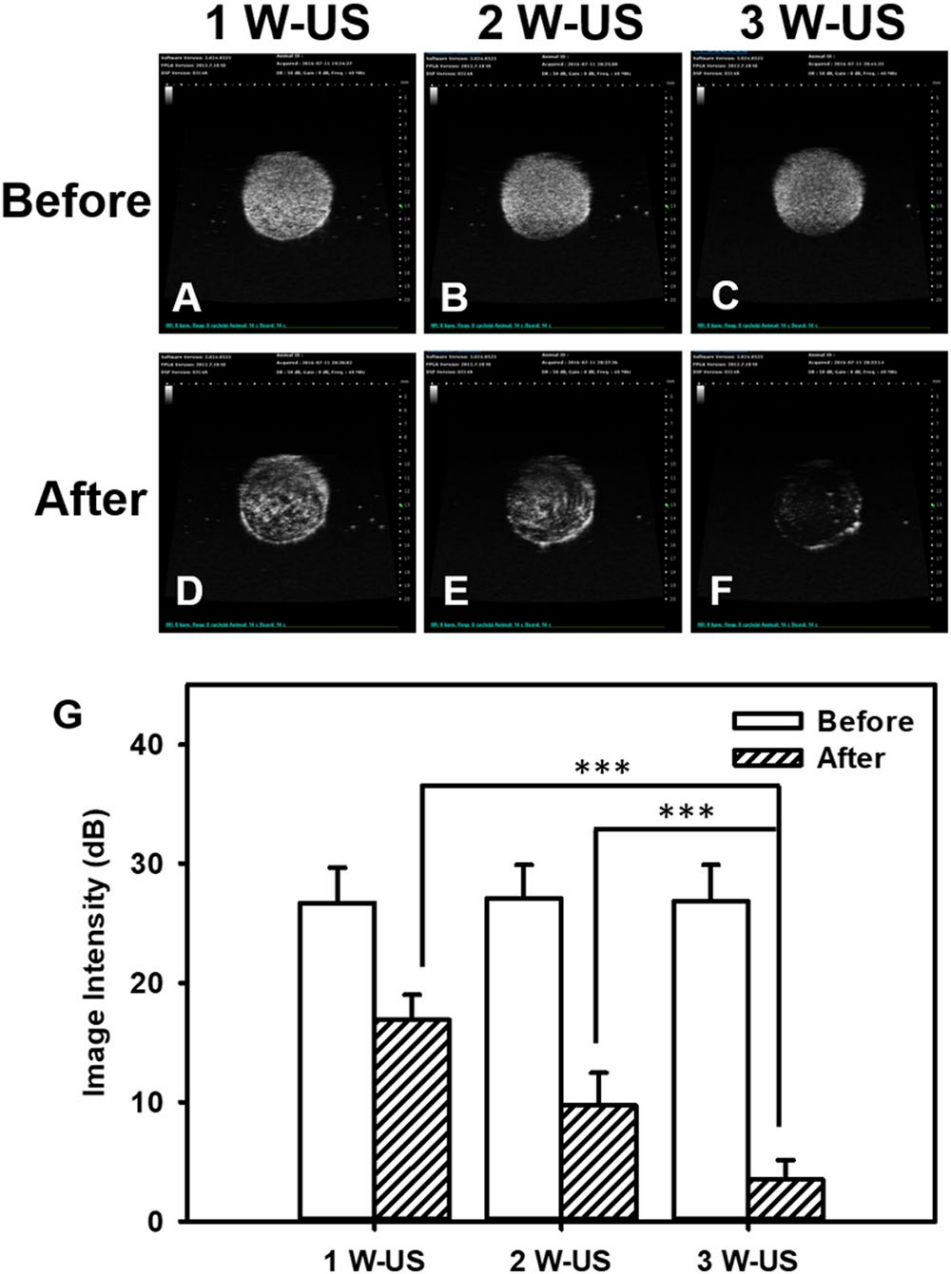
*In vitro* US images of LYMBs before (**A**–**C**) and after (**D**–**F**) US sonication across the wound dressing at 1, 2, and 3 W/cm^2^ for 1 min. (**G**) Quantification of signal intensities in (**A**–**F**) (***p < 0.001). Data are mean and SD values.

US images of 1.42 × 10^5^ LYMBs/ml without and with US sonication of the wound dressing at 1, 2, and 3 W/cm^2^ for 1 min are shown in Fig. 7(A–F), respectively, and the signal intensities in Fig. 7(A–F) are quantified in Fig. 7(G). The image intensities of the MBs in these conditions were 26.7 ± 3.0, 27.1 ± 2.8, and 26.8 ± 3.1 dB without US in Fig. 7(A–C), respectively, and 16.9 ± 2.1, 9.7 ± 2.8, and 3.5 ± 1.7 dB with US in Fig. 7(D–F), respectively, indicating that the LYMBs were significantly destroyed after the wound dressing was sonicated with US at 3 W/cm^2^ [*p* < 0.001; Fig. 7(G)]. The image intensities were decreased 36.7, 64.2, and 86.9% after 1, 2, and 3 W/cm^2^ for 1 min US irradiated.

### Measurements of penetration depth in pigskin

**Figure 8:**
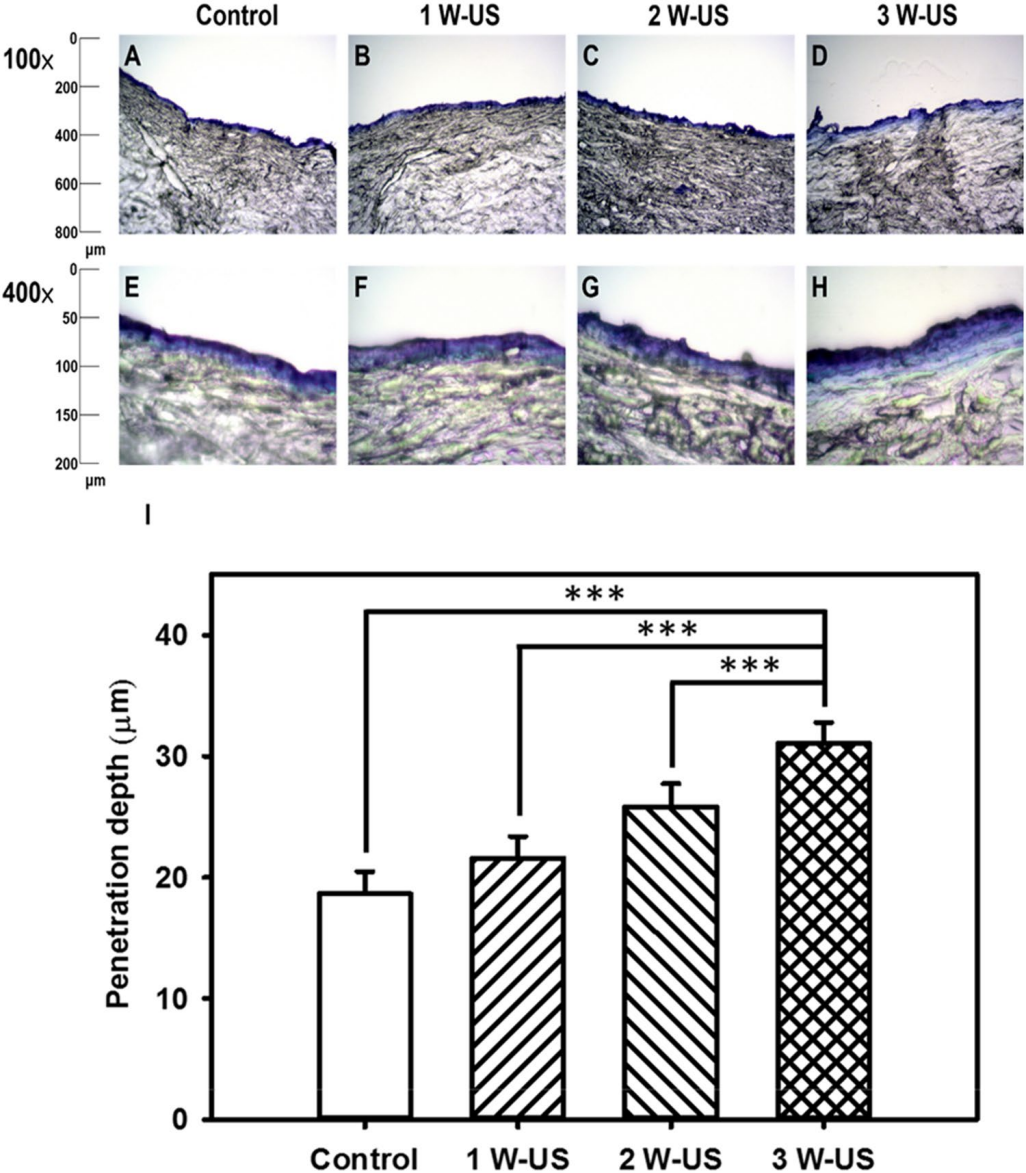
Light-microscope evaluation of pigskin samples in the control group (**A**)(**E**) and for sonication across the wound dressing at 1 W/cm^2^ (**B**)(**F**), 2 W/cm^2^ (**C**)(**G**), and 3 W/cm^2^ (**D**)(**H**) for 1 min. (**I**) Quantification of the penetration depths of Evans blue in (**A**–**H**) (***p < 0.001). Data are mean and SD values.

The pigskin samples with no treatment (group control) (Fig. 8A) and after sonicating the wound dressings containing LYMBs with US at 1 W/cm^2^ (Fig. 8B), 2 W/cm^2^ (Fig. 8C), and 3 W/cm^2^ (Fig. 8D) were then cryosectioned for light-microscopy evaluation at magnifications of ×100 and ×400 (Primo Star, Zeiss-Jena, Jena, Germany). Figure 8I quantifies the penetration depths in the four groups (*n* = 4). The degree of penetration in both the cuticle and the epidermis was significantly greater for 2 and 3 W/cm^2^ US than for 1 W/cm^2^ US (Bonferroni *p* < 0.05), and was greatest for 3 W/cm^2^ US. The overall penetration depth in group control was 18.7 ± 1.83 μm, and this increased to 21.6 ± 1.79, 25.8 ± 1.89, and 31.1 ± 1.72 μm in the groups sonicated with US at 1, 2, and 3 W/cm^2^. The penetration depth and uniformity were both greatest for 3 W/cm^2^ US, and so this condition was used for the subsequent experiments involving *in vitro S*. *aureus* colonies and *in vivo* animal treatments.

### *In vitro* treatment efficacy of EGF-LYMBs against *S*. *aureus* colonies

**Figure 9:**
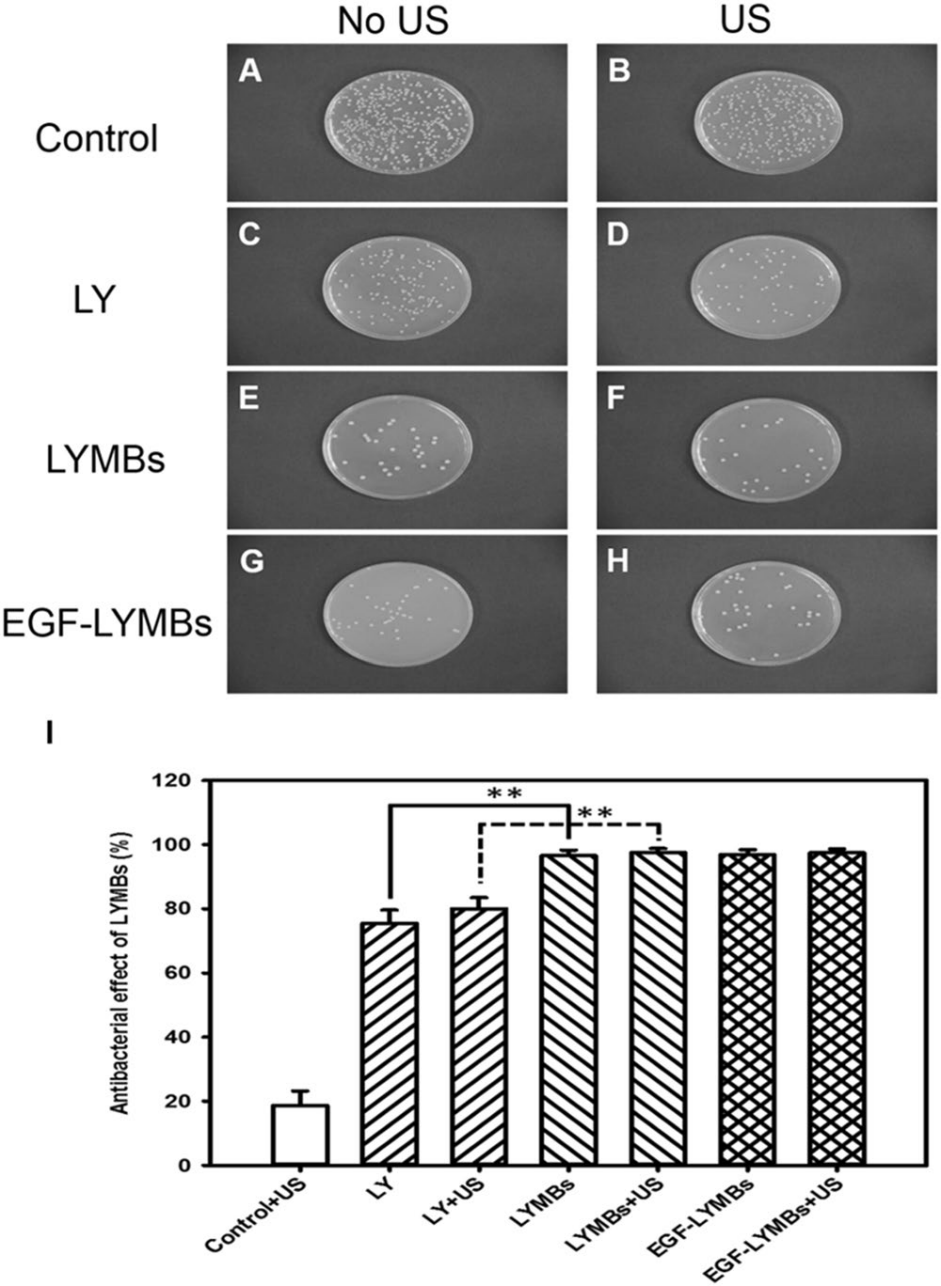
Photographs (**A**–**H**) and quantitative results (**I**) of the *in vitro* treatment efficacy in the control group (no treatment) and for the LY solution, LYMBs, and EGF-LYMBs without and with US against *S*. *aureus* colonies (**p < 0.01, *n* = 5 per group). Data are mean and SD values.

Figure 9 shows example photographs and quantitative results of the *in vitro* treatment efficacy of the control (no treatment), LY solution, LYMBs, and EGF-LYMBs without and with US against *S*. *aureus* colonies (*n* = 6). The growth of *S*. *aureus* was inhibited by 96–97% in the LYMB and EGF-LYMB groups either with or without US, and this was more effective than in the LY-solution groups either with or without US (80.03 ± 2.67% and 75.12 ± 3.04%) (*p* < 0.001).

### Animal treatments

**Figure 10:**
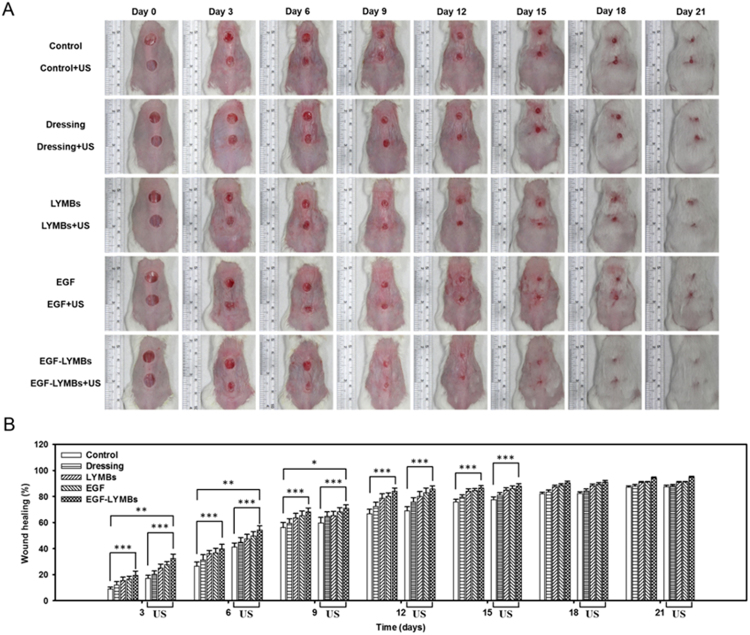
(**A**) Photographs of the wounds on mouse skin in a completely untreated animal (day 0) and in groups C, D, M, E, and EM without US (upper wounds) and with US (lower wounds) at various treatment time points. (**B**) Quantification of wound healing in the groups (*p < 0.05, **p < 0.01, ***p < 0.001).

Figure 10A shows photographs of the wounds on mouse skin in a completely untreated animal (day 0) and in groups C, D, M, E, and EM without US (upper wounds) and with US (lower wounds) at various time points after treatment. Figure 10B shows that during the first 9 days, the wound healing increased more (*p* < 0.05) in the groups with US than in those without US. At day 6, the wound healing for the five mice was better in group US + EM (54.28 ± 3.26%) than in groups US + E (49.73 ± 3.22%), US + M (47.31 ± 3.59%), US + D (44.83 ± 3.65%), US (41.16 ± 3.10%), and C (26.36 ± 3.34%). At that time point there were obvious significant differences (*p* < 0.01) between group US + EM and the other four US groups. At days 9 and 12 there were significant differences (*p* < 0.05) between groups US + EM and US + E. At day 15 there was no longer a significant difference (*p* > 0.05) between these two groups, but the wound healing was still better (*p* < 0.05) in group US + EM than in groups US + M, US + D, and US. At day 18 the wound healing had reached a plateau, and there were no significant differences (*p* > 0.05) between the groups without US (C = 87.14 ± 1.03%, D = 87.85 ± 1.25%, M = 90.68 ± 0.84%, E = 91.02 ± 0.52%, and EM = 94.10 ± 0.70%) and with US (US + C = 87.48 ± 1.08%, UD + D = 88.12 ± 1.16%, US + M = 90.66 ± 0.77%, US + E = 91.02 ± 0.44%, and US + EM = 94.86 ± 0.67%).

### Histochemistry

**Figure 11:**
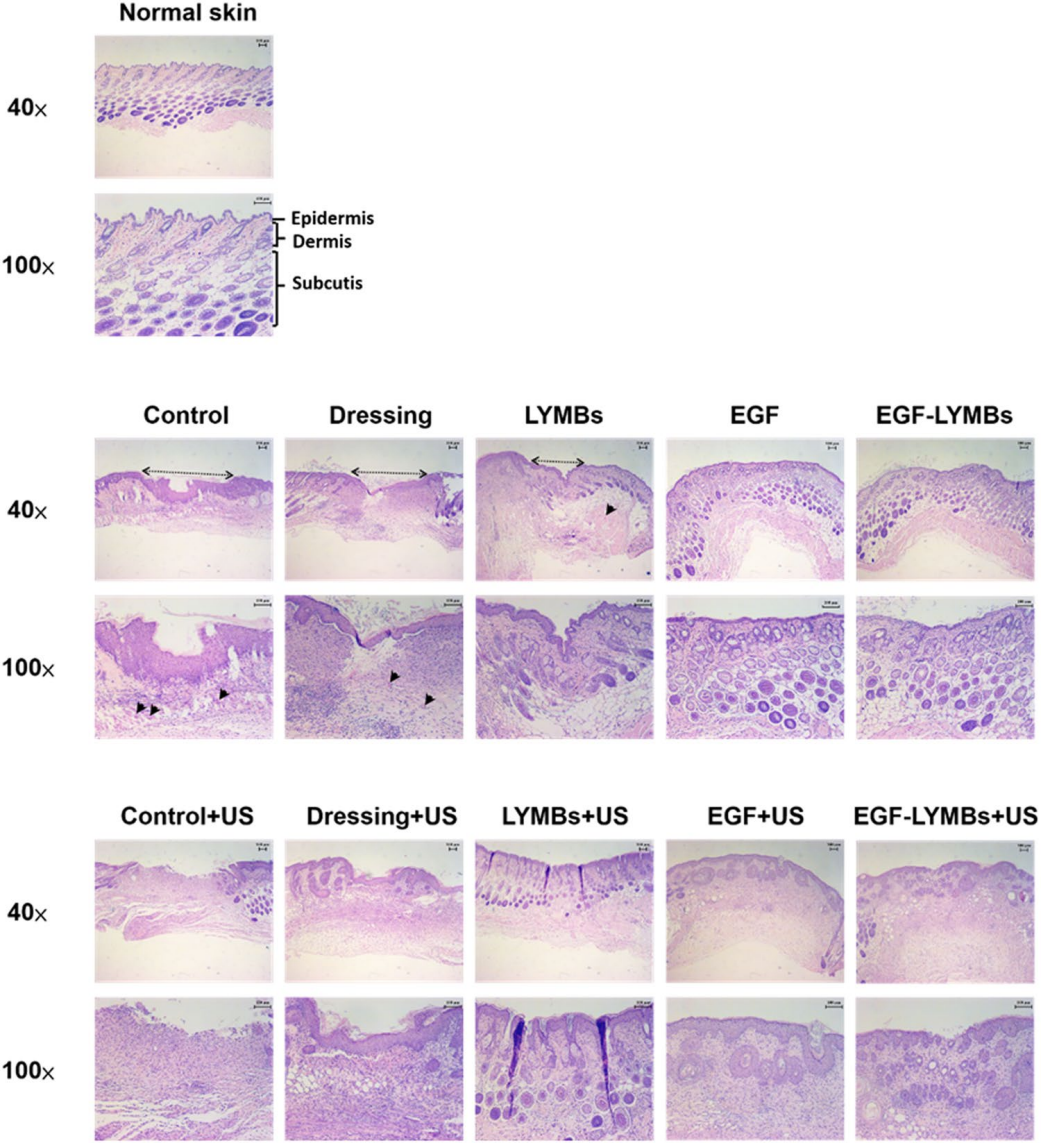
Histology of normal skin (upper row), wound area with treatments in various groups (middle row), and wound area with US treatment in various groups (lower row) on day 21 after treatment, for staining with hematoxylin and eosin. Photomicrographs of wound tissues at different magnifications are shown in the pairs of rows. Arrowheads show the different levels of neovascularization in various groups.

The histological sections stained with hematoxylin and eosin exhibited marked morphological differences in both uninjured skin (upper row in Fig. 11) and wounds in groups C, D, and M (middle row in Fig. 11). Groups E and EM showed a histological structure that was most similar to that of normal skin. At 21 days after treatment, groups E and EM groups showed almost complete epithelialization in the wound area, whereas groups C, D, and M still exhibited gaps of several millimeters between the epithelial tips. Inflammatory cell infiltration did not differ significantly between groups E and EM. More collagen and fibroblasts were evident in groups E and EM treated with US (lower row) and without US (middle row) that received EGF. The proliferation of blood capillaries and angiogenesis were significantly more pronounced in groups treated with US.

**Figure 12:**
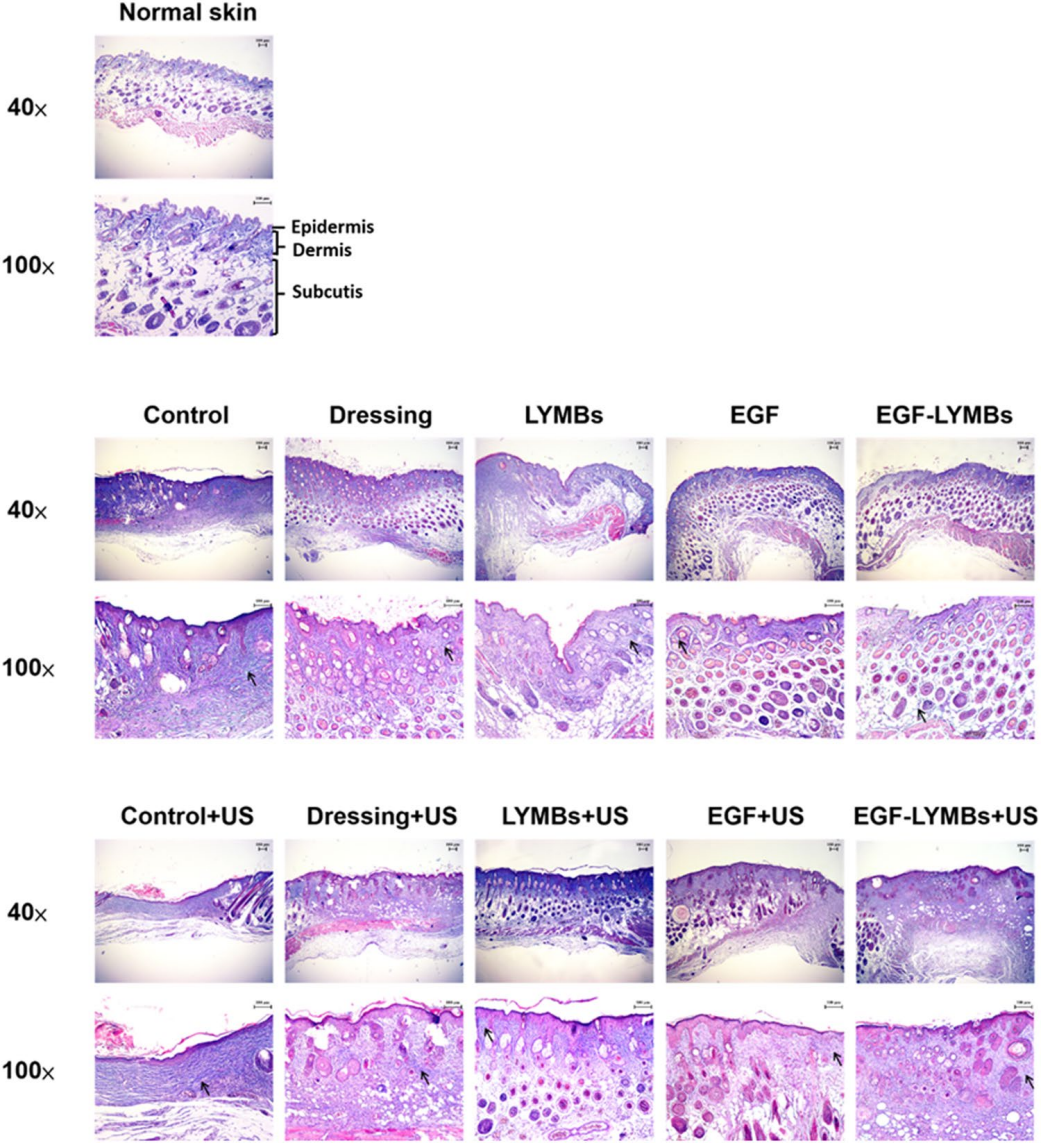
Histological sections of healed wounds on day 21 after treatment stained with Masson’s trichrome. The wounds are shown at different magnifications in the pairs of rows. Arrows show the different levels of collagen deposition, represented by the intensity of the green or blue color.

Figure 12 shows the results of Masson’s trichrome staining of the wound after 21 days. The presence of collagen and fibroblasts was similar in the non-US-treated (middle row) and US-treated (lower row) groups, with a higher density of collagen and more fibroblast cells than in normal skin (upper row). The non-US-treated groups (middle row) showed significantly less collagen and fewer fibroblasts and blood capillaries in comparison with the US-treated groups (lower row).

## Discussion

LY is a hydrolase commonly found in infected skin wounds, and it has previously been found that the formation of a loosely polyelectrolyte architecture caused rapid EGF diffusion in an infected wound that had been simulated by adding LY to the release medium^36^. LY is a small (4.5 nm × 3 nm × 3 nm) protein consisting of 129 amino acid residues with a molecular weight of 14,300 and an isoelectric point of 11.1^37^, which means that LY is always positively charged under physiological conditions.

In the present study, EGF-LYMBs were assembled via the electrical adsorption of EGF onto LYMBs, added to wound dressings, and combined with US with the aim of enhancing wound healing. This study found that an appropriate amount of EGF resulted in greater EGF encapsulation. Table 1 indicates that EGF-LYMBs with EGF present at 100 µg/ml had the highest loading efficiency (19.40 ± 0.04%) of EGF onto LYMBs, and that this did not vary with the amount of EGF (e.g., EGF = 200 µg/ml), which may be due to the saturation of EGF loading in the LYMBs causing a shorter survival time (LYMB disruption). These EGF-LYMB shells were washed away by the washing procedure, resulting in an encapsulation efficiency of only 9.74 ± 0.06% in this group. Moreover, ultrasound frequency of 1 MHz was chosen to enhance LYMBs and EGF-LYMB100s cavitation in wound healing due to the diameters of the LYMBs and EGF-LYMB100s were 2680 ± 210 and 3780 ± 180 nm, respectively. A previous study in the literature shows that the nucleating bubbles are small (the radius is ~3 μm at 1 MHz), and can nucleate within the stratum corneum (SC), giving rise to some disruption of the ordered SC structure^31^.

An acidic milieu is present on the skin surface under normal circumstances, and its pH value varies from 4 to 6 depending on the anatomical location and age of the person^38^. An *in vitro* investigation found that while the application of US energy can enhance drug release, using a low pH value of 5.0 can also increase the efficiency of EGF release from LYMBs. Although the enzyme activity of LY (isoelectric point = 9.32) is optimal at pH 5.0 and decreases above and below this pH, the affinity of EGF decreases at pH values lower than 5.0^39,40^. The swelling properties of EGF in low pH may lead to unstable conjugation between EGF and LYMBs and thereby increase the EGF released from LYMBs. This property can be utilized to improve the delivery of EGF into the wound since the pH of an acute wound is typically around 5.5^38^. However, chronic wounds and infected wounds with a high bacterial load can exhibit pH values above 7.3^41^. The pH environment of chronic wounds might influence the EGF release from LYMBs in a wound dressing.

According to the results of the present study, LYMBs added to a wound dressing did not affect the survival of LYMBs (Fig. 5E). However, the survival of LYMBs in a wound dressing and the efficacy of drug penetration were affected by US treatments. This efficacy can be controlled by optimizing the MB conditions, such as their concentration. The high-frequency US phantom imaging (Fig. 7) showed that applying 3 W/cm^2^ US to an LYMB phantom across the wound dressing resulted in the significant destruction of LYMBs (86.9%). It is consistent with the penetration depth and uniformity in pigskin (Fig. 8) both being greatest for 3 W/cm^2^ US, and so this condition was used for all of the experiments involving *in vitro S*. *aureus* colonies and *in vivo* animal treatments.

Figure 9 shows that *S*. *aureus* was inhibited more effectively in the LYMB groups than in the LY-solution group for LY at the same concentration. These results are consistent with those obtained in our previous study when combining LYMBs with US to significantly inhibit the growth of *Propionibacterium acnes*, since the phagocytosis of MBs increases the cytotoxicity^25,42^. In the present study, US sonication at a power density of 3 W/cm^2^ did not markedly suppress the growth of *S*. *aureus* either in the LYMB or EGF-LYMB group, due to the inhibition reaching saturation. Combining US at a power density of 3 W/cm^2^ with the LY solution suppressed the growth of *S*. *aureus* slightly. The results showed that the EGF coating did not affect the antibacterial effect of LYMBs and that the *in vitro* antibacterial effect was not affected by the US.

In the animal experiments, the wound healing was better in group US + EM (54.28 ± 3.26%) than in the other groups and was close to that in group US + E during days 9 to 18. The histological observations in Figs 11 and 12 reveal that groups E and EM showed almost complete epithelialization in the wound area, whereas other groups still contained gaps of several millimeters between the epithelial tips. This is due to topical application of EGF, which can enhance epidermal regeneration in patients with ulcers, and it is also widely accepted that EGF can stimulate epithelialization in human wound repair. In Fig. 11, the neovascularization only can be observed in C, D, and M groups (arrowheads point to the neovascularization lesion). The early presence of VEGF and basic fibroblast growth factor stimulated vascular permeability and the proliferation of endothelial cells^43^. Almost complete epithelialization in the wound areas were observed in all of the US-treated groups in the present study.

The US-induced improvements in neovascularization and wound healing in excisional wounds were investigated in the present study. The obtained results confirm that combined treatment with US and EGF-LYMBs can reduce the duration of wound healing, inhibit *S. aureus*, promote neovascularization and wound healing, and significantly improve the prognosis of a wound.

## Conclusion

This study has produced a new integrated wound healing platform for enhancing and monitoring the delivery of EGF to a wound area by utilizing US-mediated EGF-LYMB cavitation. EGF-LYMBs were assembled by the electrical adsorption of EGF onto LYMBs, and the release of EGF from LYMBs was increased at pH 5.0. The antimicrobial efficacy when using LYMBs or EGF-LYMBs reached 96–97%, which was higher than in LY-solution groups with or without US. In the *in vitro* experiments, the wound healing was better in group US + EM than in the other groups, and EGF-LYMBs combined with US were found to significantly reduce the duration of wound healing, promote neovascularization and wound healing, and improve the wound prognosis. Moreover, in contrast to standard treatment, almost complete epithelialization in the wound areas was observed in all US-treated groups.
